# Supplementary material for: Over 1000 tools reveal trends in the single-cell RNA-seq analysis landscape
Source: Genome Biol. 2021 Oct 29;22:301. doi: 10.1186/s13059-021-02519-4 (PMC8555270; doi:10.1186/s13059-021-02519-4)
Supplement: Supplementary file 1 — Additional file 1: Supplementary Figure 1. Delay in adding tools to the database. Supplementary Figure 2. Fit of the number of tools over time. Supplementary Figure 3. Platform proportions by category. Supplementary Figure 4. Number of categories per tool. Supplementary Figure 5. Dependencies between tools. Supplementary Figure 6. Correlations between publications and tools metrics. Supplementary Table 1. Coefficients for publications models. Supplementary Table 2. Coefficients for tools models. [file 13059_2021_2519_MOESM1_ESM.pdf]

Supplementary information
Over 1000 tools reveal trends in the single-cell RNA-seq analysis landscape

Table of contents

Table of contents **1**

Supplementary figures **2**

Supplementary Figure 1: Delay in adding tools to the database 2
Supplementary Figure 2: Fit of the number of tools over time 3
Supplementary Figure 3: Platform proportions by category 4
Supplementary Figure 4: Number of categories per tool 5
Supplementary Figure 5: Dependencies between tools 6
Supplementary Figure 6: Correlations between publications and tools metrics 7

Supplementary tables **8**

Supplementary Table 1: Coefficients for publications models 8
Supplementary Table 2: Coefficients for tools models 8

Supplementary figures

Supplementary Figure 1: Delay in adding tools to the database

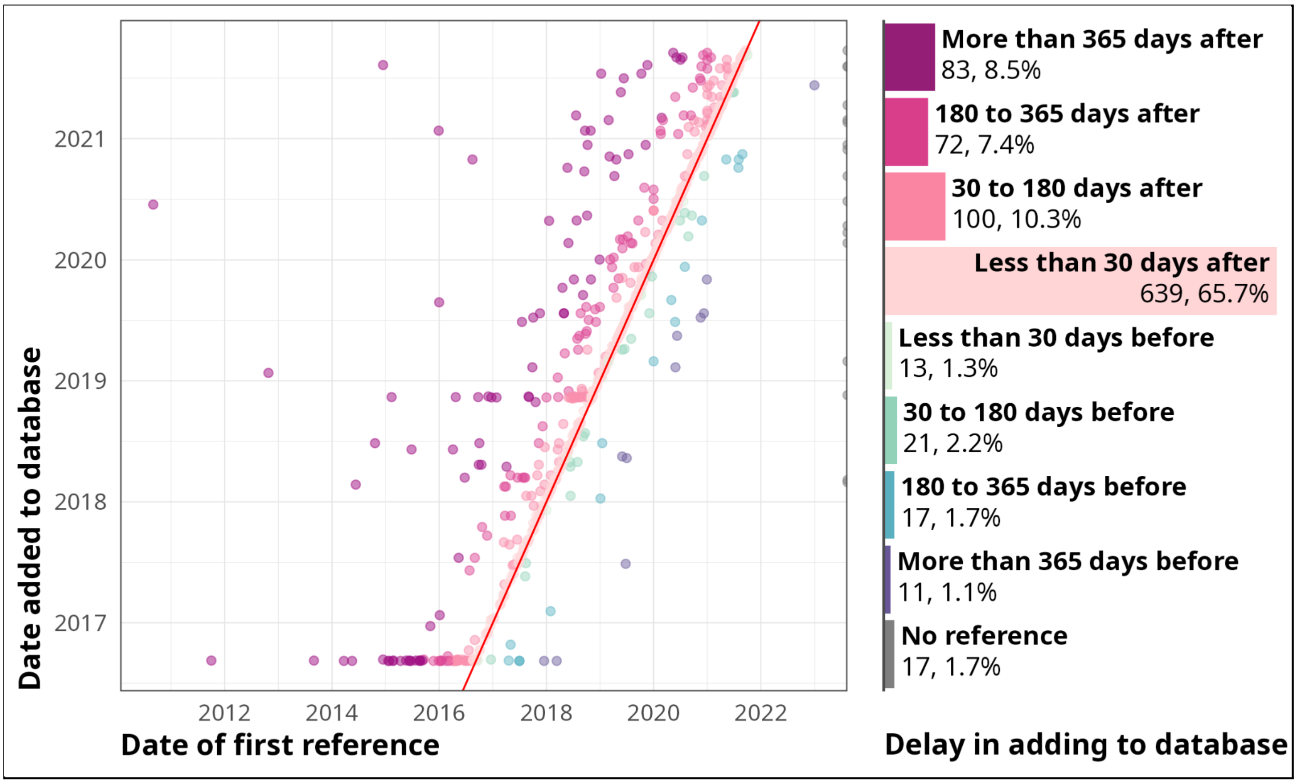

Figure S1: Delay in adding tools to the scRNA-tools database. The scatter plot shows the date tools were added to the database against the date of the first reference (preprint or publication). Points are grouped and coloured according to how many days before or after the reference data they were added to the database. The bar chart shows the proportions for these groups with around two-thirds or of tools added less than 30 days after a reference becomes available.

Supplementary Figure 2: Fit of the number of tools over time

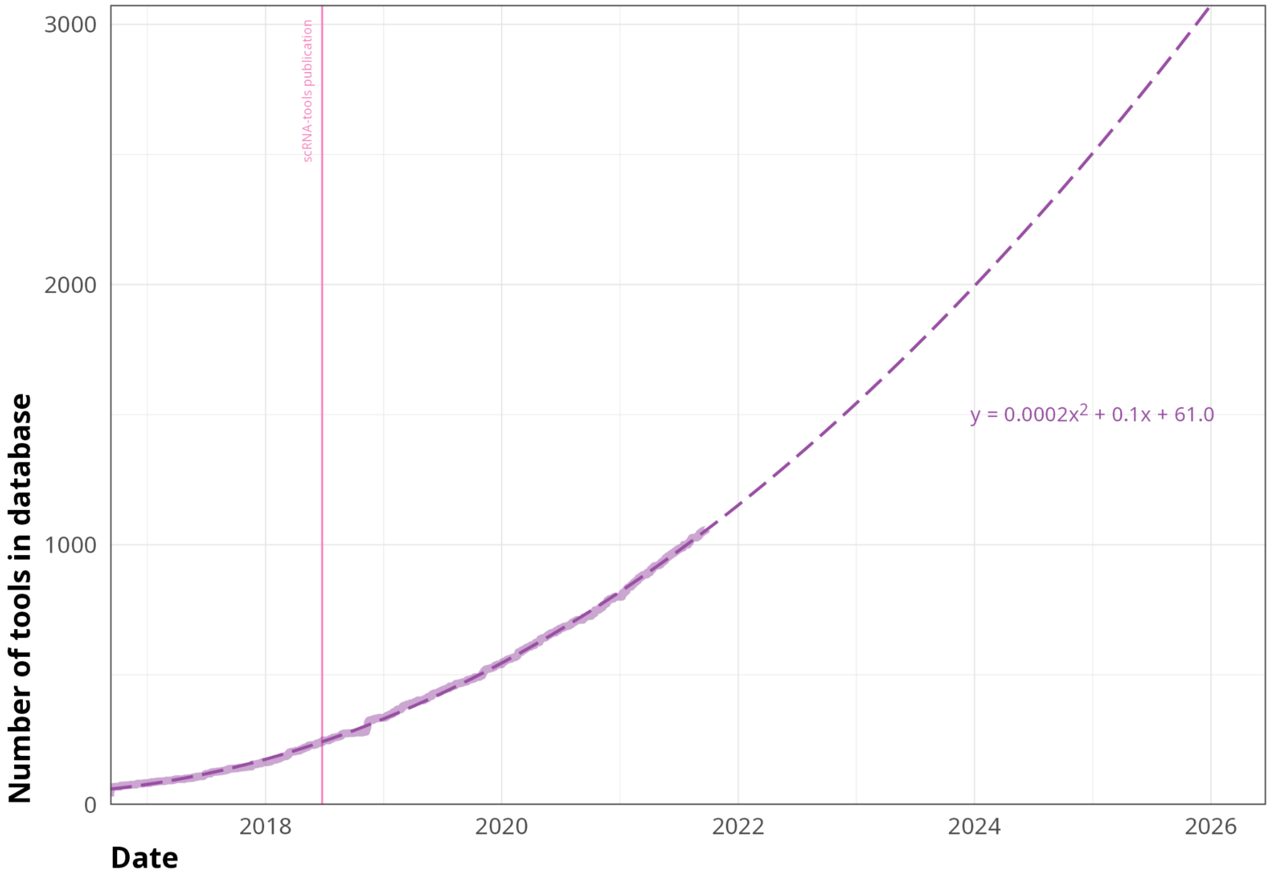

Figure S2: Line plot of the number of tools in the scRNA-tools database over time with quadratic fit (dashed line, $y = 0.0002x^2 + 0.1x + 61.0$). This trend predicts more than 1500 tools by the end of 2022 and more than 3000 by the end of 2025. The vertical pink line shows the date of the original scRNA-tools publication.

Supplementary Figure 3: Platform proportions by category

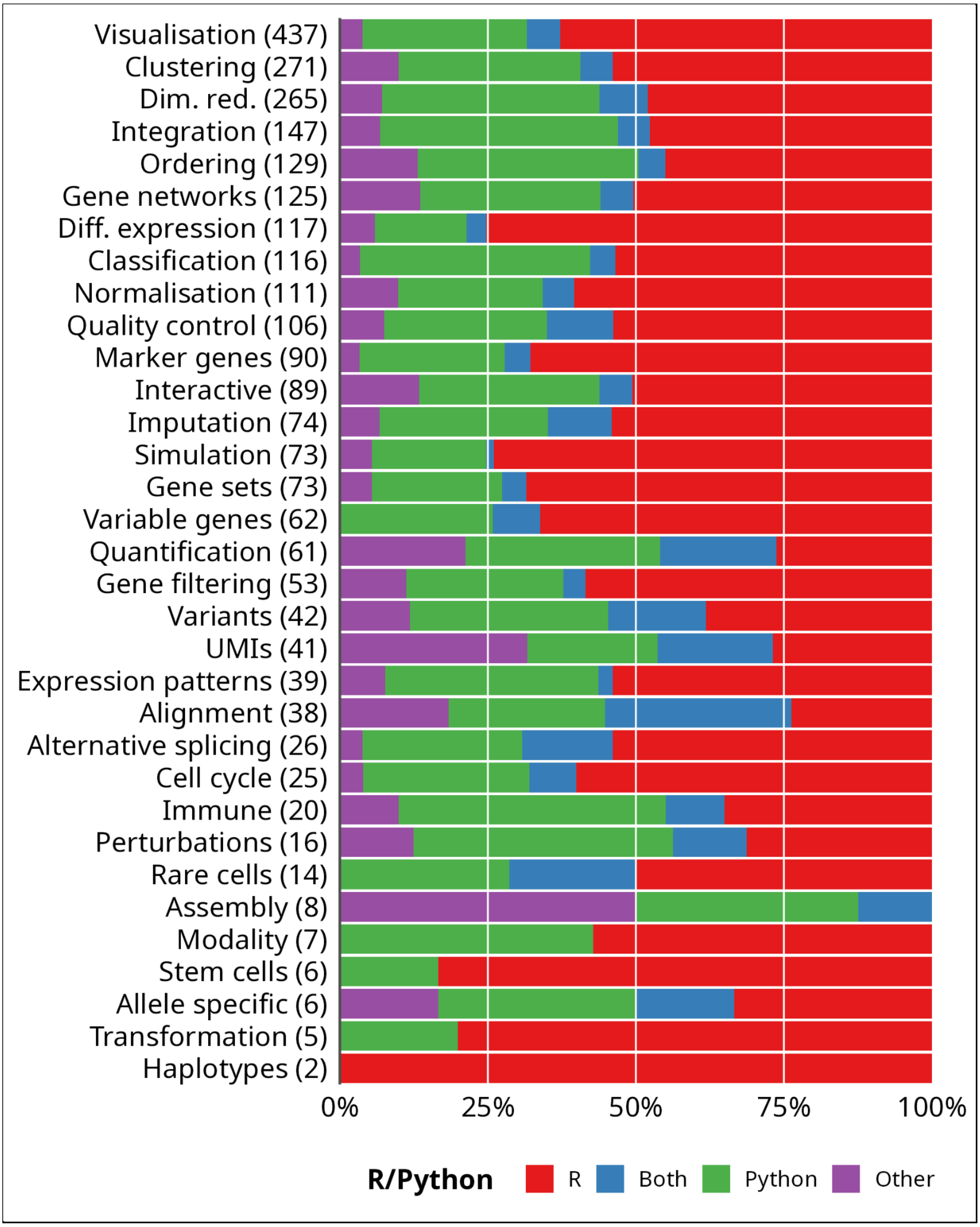

Figure S3: Stacked bar chart of proportions of tools in each analysis category built using R, Python or other platforms. Numbers in brackets show the number of tools belonging to each category.

Supplementary Figure 4: Number of categories per tool

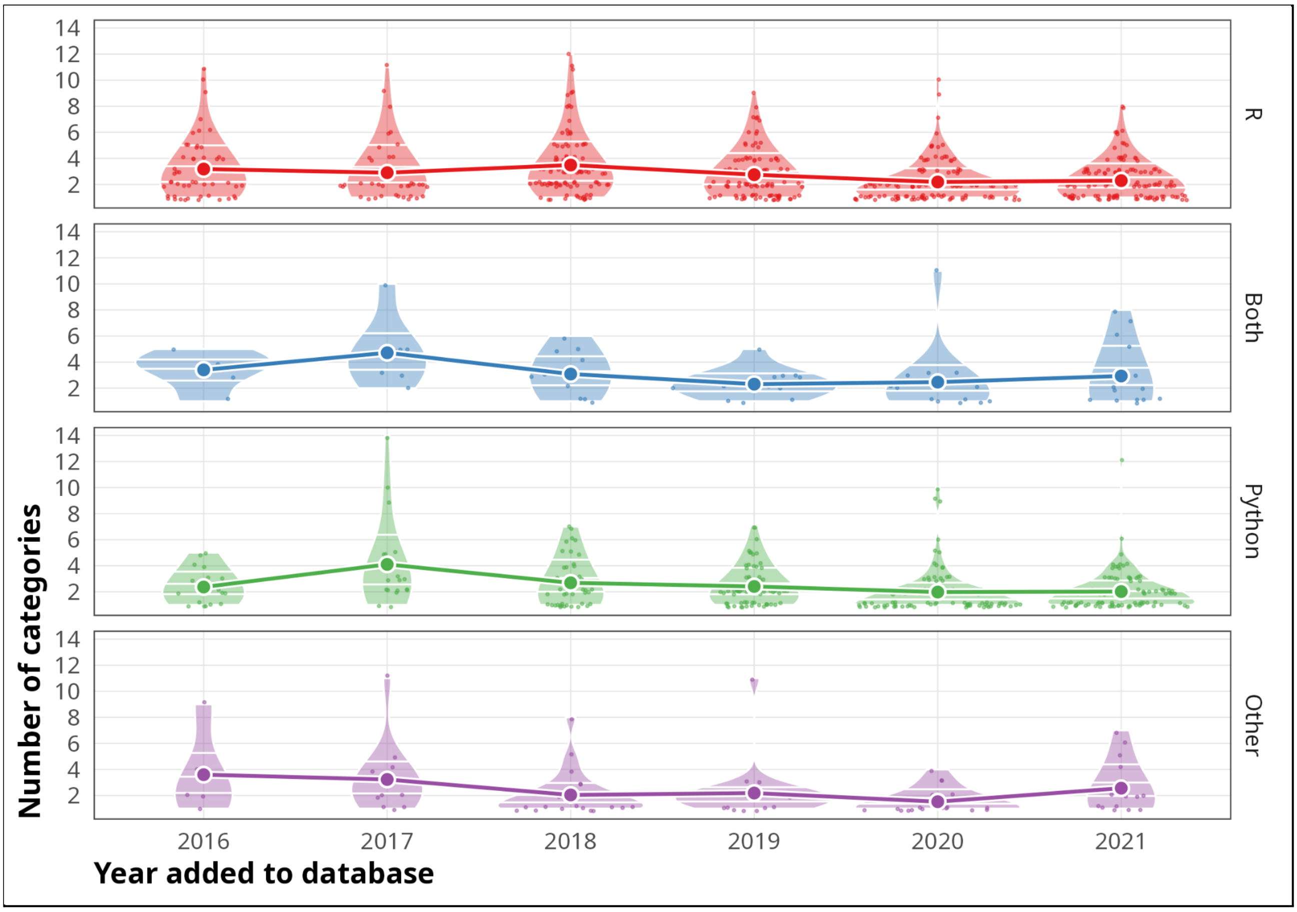

Figure S4: Number of categories per tool. Violin plots are shown divided by year and major platforms with points representing individual tools. Large points connected by lines indicate the trend in the mean number of categories per tool over time.

Supplementary Figure 5: Dependencies between tools

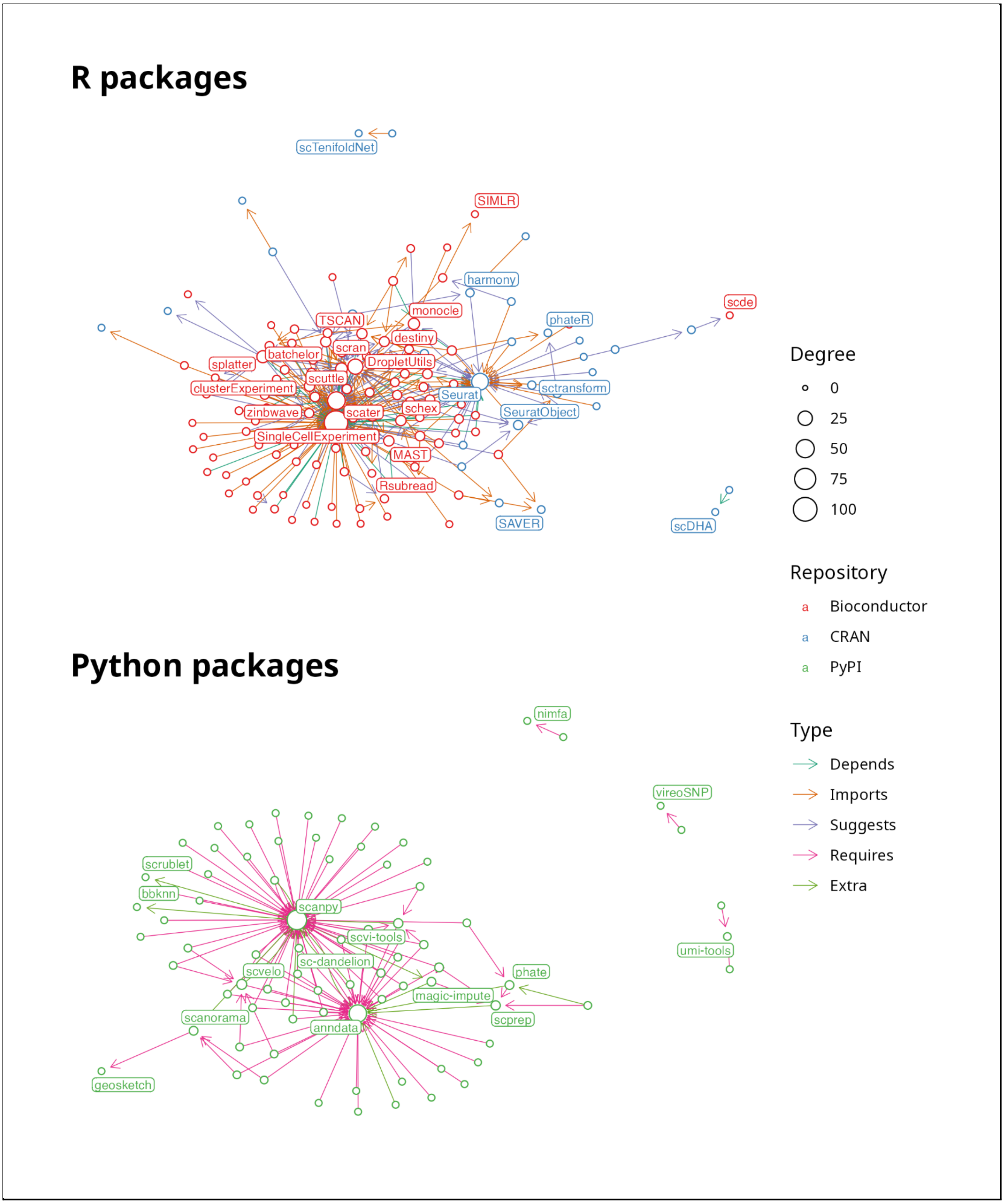

Figure S5: Dependency graphs for R and Python packages available from public software repositories. Nodes in the graph represent packages and edges represent dependencies. Node size shows degree and node colour indicates software repository. Edge colour indicates dependency type. Nodes with high PageRank centrality scores are labelled.

Supplementary Figure 6: Correlations between publications and tools metrics

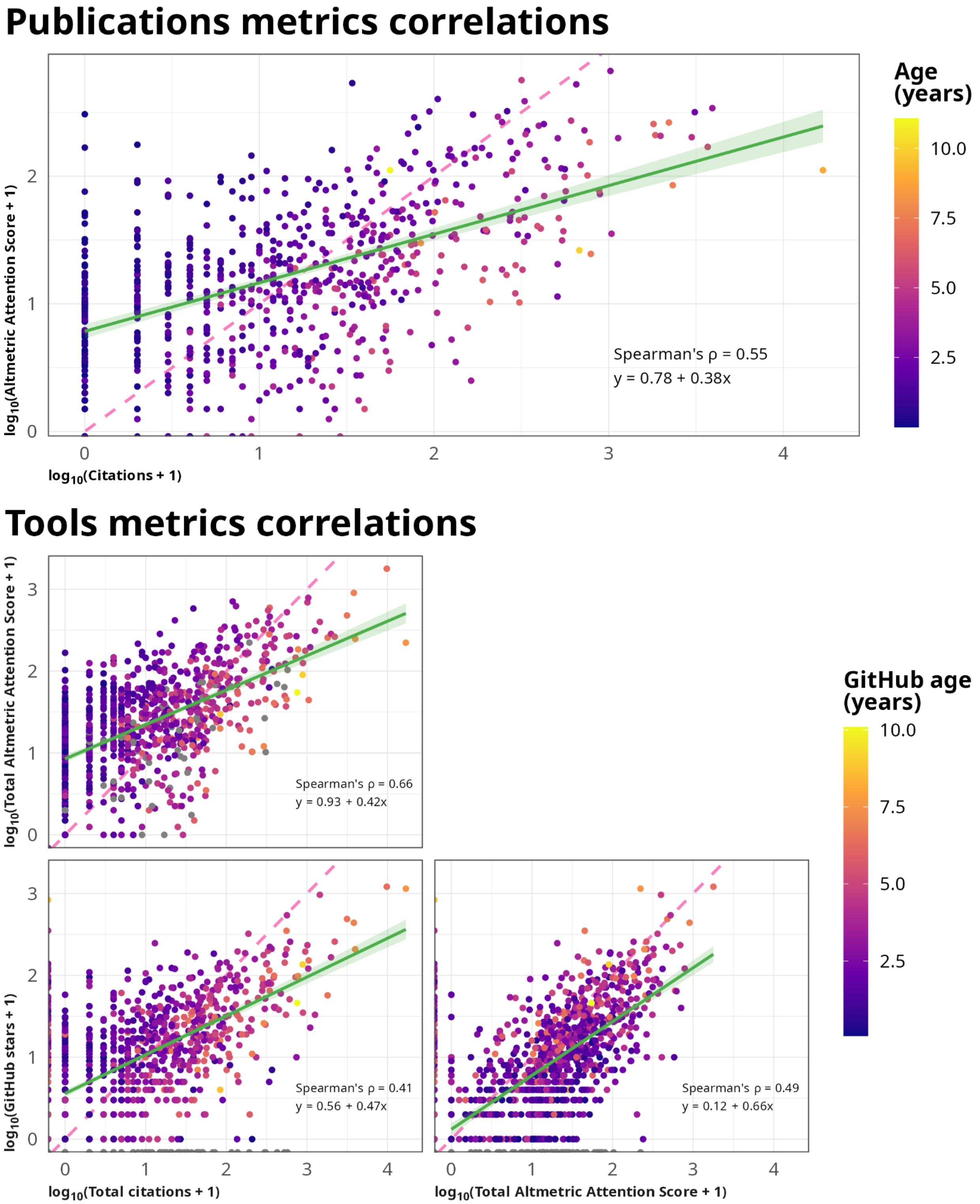

Figure S6: Relationship between publications (top) and tools (bottom) metrics. Scatter plots show the relationship between each pair of metrics on a log scale with each point and individual publication or tool coloured by age. Dotted pink lines show the $y = x$ line and green lines show a linear fit with a 95 percent confidence interval. Annotations give equations for the linear fit and Spearman's correlation coefficient. Publications metrics are citations and Altmetric Attention Score, tools metrics are total citations, total Altmetric Attention Score and GitHub stars.

Supplementary tables

Supplementary Table 1: Coefficients for publications models

Table S1: Coefficients and 95 percent confidence for publications models

Term	Citations	AAS
(Intercept)	-3.62 (-4.26, -2.97)	-0.09 (-0.80, 0.62)
$\log_2$(Num references + 1)	0.34 (0.24, 0.44)	0.33 (0.21, 0.44)
$\log_2$(Num authors)	0.49 (0.37, 0.62)	0.46 (0.33, 0.60)
Has preprint	0.82 (0.58, 1.06)	0.64 (0.39, 0.90)
Years (1st degree)	6.25 (5.56, 6.93)	0.75 (0.03, 1.47)
Years (2nd degree)	12.51 (11.34, 13.67)	3.87 (2.65, 5.08)
Years (3rd degree)	9.59 (7.86, 11.31)	4.00 (2.21, 5.79)

Supplementary Table 2: Coefficients for tools models

Table S2: Coefficients and 95 percent confidence for tools models

Term	Total citations	Total AAS	GitHub popularity
(Intercept)	-1.16 (-1.81, -0.51)	1.23 (0.63, 1.82)	-1.48 (-2.03, -0.94)
Has repository	0.19 (-0.08, 0.47)	0.27 (0.02, 0.52)	0.01 (-0.22, 0.24)
Has license	0.09 (-0.21, 0.39)	0.30 (0.02, 0.58)	0.45 (0.20, 0.71)
$\log_2$(Contributors + 1)	0.52 (0.35, 0.69)	0.57 (0.41, 0.72)	0.89 (0.75, 1.04)
Platform (R)	0.09 (-0.34, 0.53)	-0.14 (-0.54, 0.26)	0.24 (-0.13, 0.60)
Platform (Python)	0.21 (-0.25, 0.67)	0.12 (-0.29, 0.54)	0.78 (0.40, 1.17)
Platform (Both)	0.56 (-0.06, 1.18)	0.20 (-0.36, 0.76)	0.85 (0.33, 1.36)
Preprints	0.20 (-0.01, 0.41)	0.95 (0.75, 1.14)	0.51 (0.33, 0.68)
Publications	1.48 (1.25, 1.72)	0.76 (0.55, 0.98)	0.35 (0.15, 0.55)
Years (1st degree)	4.70 (4.03, 5.38)	0.68 (0.07, 1.29)	1.66 (1.10, 2.23)
Years (2nd degree)	5.79 (4.37, 7.21)	1.66 (0.37, 2.96)	5.09 (3.90, 6.28)
Years (3rd degree)	5.54 (3.88, 7.20)	0.81 (-0.70, 2.32)	2.84 (1.45, 4.23)
