# Supplementary material for: Over 1000 tools reveal trends in the single-cell RNA-seq analysis landscape
Source: Genome Biol. 2021 Oct 29;22:301. doi: 10.1186/s13059-021-02519-4 (PMC8555270; doi:10.1186/s13059-021-02519-4)
Supplement: Supplementary file 2 — Additional file 2. Review history. [file 13059_2021_2519_MOESM2_ESM.docx]

Review History

**First round of review**

**Reviewer 1**

**Comments to author:**

Here, Zappia and Theis present an overview of the scrna-tools.org database on occasion of it surpassing 1,000 tools. Having good quality data from 2017, the authors are in a unique position to survey this rapidly evolving field. Overall, I find the manuscript well-written and it is likely to be of high interest to the community as it provides a bird's eye perspective on the computational tools that have been developed.

I have the following suggestions that I hope can strengthen the manuscript further:

1. On p3, line 58 the authors discuss the differences between packages from software repositories and those that are available from GitHub. I was wondering if it would be possible to test if there is a difference? Similarly to what they do in Fig 3, would it be possible to develop a quantitative model to test if difference in installation procedure influences the popularity of a tool?

2. I was wondering if the authors could elaborate on how new categories are added? I recognize that this is at the end of the day a call from the maintainers of the db, but it would be helpful if you could outline the principles you are using and comment on how frequently new terms have been added in the past.

3. P6, line 41-42. This feels like speculation and would fit better in the discussion than in the results.

4. The results in fig 3 showing the coefficients from the linear model I find quite interesting. However, I was wondering if it would be meaningful to also include variables such as the number of commits, the number of issues and the number of contributors. In my mind, they would be indicators of an active user community and I would like to see how that is related to tool popularity. For the tools in my own lab I always like to think that more GitHub activity is better and I am curious to see if there is statistical support for this.

5. It is valuable for developers to know what process you are using for adding tools to the db (and that one can be pro-active and add them oneself!). Again, if you have the data I was wondering if you could include some numbers on the lag times between when a tool is published and when it gets included into your db?

6. I think that the temporal trends on tool types is very fascinating and informative when thinking about how the field is evolving. I would like to propose an additional means of visualization here: word clouds. Would it be possible to take annual snapshots of the data used in fig 2c and represent the words from the abstracts as word clouds?

7. For fig S1 it would be helpful to add numbers on the right to indicate the total number of tools in each category.

8. Fu and Hughey point out that in their dataset altmetric score and citations are only weakly correlated. Could the authors calculate this correlation for their dataset?

9. The other aspect that I was curious about the models in Fig 3 is the % of variance explained. Could you report this quantity to give a better indication of how well the models explain the data?

**Authors Response**

**Point-by-point responses to the reviewers’ comments:**

Reviewer #1

Here, Zappia and Theis present an overview of the scrna-tools.org database on occasion of it surpassing 1,000 tools. Having good quality data from 2017, the authors are in a unique position to survey this rapidly evolving field. Overall, I find the manuscript well-written and it is likely to be of high interest to the community as it provides a bird's eye perspective on the computational tools that have been developed.

*Thank you for taking the time to review our manuscript and for your kind words about our work. We have addressed your comments below.*

I have the following suggestions that I hope can strengthen the manuscript further:

1. On p3, line 58 the authors discuss the differences between packages from software repositories and those that are available from GitHub. I was wondering if it would be possible to test if there is a difference? Similarly to what they do in Fig 3, would it be possible to develop a quantitative model to test if difference in installation procedure influences the popularity of a tool?

*The vast majority (over 90 percent) of tools have an associated GitHub repository, including the ~35 percent of tools that can be installed from a central software repository (CRAN/Bioconductor/PyPI). Because this number is so high it is difficult to test differences between those with/without GitHub. In the modelling in Figure 3 we include a “Has repository” variable which captures whether or not a tool can be installed from a central repository. In the original analysis we saw a small positive effect on the various metrics from this but that is no longer the case after updating the model (more details in the response to point 9 below including changes to the text).*

2. I was wondering if the authors could elaborate on how new categories are added? I recognize that this is at the end of the day a call from the maintainers of the db, but it would be helpful if you could outline the principles you are using and comment on how frequently new terms have been added in the past.

*In general, we can add a new category when it is required to capture an aspect of analysis that is not currently represented by the existing categories but we do not have clear guidelines for when this is the case. Currently, the only new category we have added since the original publication is the “Perturbations” category but there is a list of potential new categories on this GitHub issue https://github.com/scRNA-tools/scRNA-tools/issues/90. The main barrier to adding new categories is the time required to re-curate tools currently available in the database and we plan to ask for help from the community to do this going forward. These points have been described in the methods section.*

*“We believe that the categorisation of tools according to the tasks they perform is an important feature of the scRNA-tools database. The current categories have been designed to cover the main stages of a standard scRNA-seq analysis workflow but may not fully represent alternative or new analysis approaches. When the need for a new category becomes apparent it can be added to the database as we have done for perturbations. Other categories under consideration include cell-cell interactions, infrastructure, time series and cancer. The major bottleneck to adding new categories is the time required to re-categorise tools already in the database and we welcome support from the community for this task.”*

*We have also added a sentence to the conclusion stating that we encourage suggestions for new categories.*

*“This includes suggestions for new categories to capture aspects of analysis that are not currently represented.”*

3. P6, line 41-42. This feels like speculation and would fit better in the discussion than in the results.

*Thanks, this sentence has been moved down as requested.*

4. The results in fig 3 showing the coefficients from the linear model I find quite interesting. However, I was wondering if it would be meaningful to also include variables such as the number of commits, the number of issues and the number of contributors. In my mind, they would be indicators of an active user community and I would like to see how that is related to tool popularity. For the tools in my own lab I always like to think that more GitHub activity is better and I am curious to see if there is statistical support for this.

*As suggested, we have explored adding more GitHub metrics to the models for tools however there is an issue of causality. We feel that it is unlikely that GitHub activity leads to more usage which leads to more citations but rather that some property of the tool leads to more users which leads to both more GitHub activity and more citations. Of course, there are some cases where a lack of responsiveness on GitHub leads to a tool not being used even if there is interest in the method but these are exceptions to the general trend. One exception we have made is to include the number of GitHub contributors in the model to mirror the number of authors in the models for publications, however, it still comes with some of the same issues which we have described in the text:*

*“The other significant predictor for all three metrics was the number of GitHub contributors. While this is similar to the number of authors of a publication, an important distinction is that members of the community can contribute code over time (unlike publication where the number of authors is fixed). This makes it difficult to establish the direction of the relationship to the metrics modelled here, i.e. do more contributors lead to better tools that get more citations and stars or do tools that are already highly used attract more contributors? While including GitHub contributors result in models with better fits it is possible that it also masks the smaller effects of other predictors such as the availability of tools from a software repository.”*

5. It is valuable for developers to know what process you are using for adding tools to the db (and that one can be pro-active and add them oneself!). Again, if you have the data I was wondering if you could include some numbers on the lag times between when a tool is published and when it gets included into your db?

*Thanks for the suggestion. We have included a supplementary figure showing the delay between the first reference for a tool and when it was added to the database. Around two-thirds of tools are added to the database within 30 days of the first reference (and most of those are added within 10 days). This is described in the text:*

*“Most tools are added to the database within 30 days of the first preprint or publication (Additional File 1: Figure S1)”*

6. I think that the temporal trends on tool types is very fascinating and informative when thinking about how the field is evolving. I would like to propose an additional means of visualization here: word clouds. Would it be possible to take annual snapshots of the data used in fig 2c and represent the words from the abstracts as word clouds?

*Thank you for the suggestion. We have added a new panel to Figure 2 showing word clouds by year. For each year we show the 20 words with the biggest change in the proportion of abstracts compared to the previous year.*

*“Figure 2D shows this the same data as a series of word clouds by year. The twenty words with the biggest change in proportion compared to the previous year are shown with colour indicating the change and size indicating the proportion in that year. These terms are less specific than those found by looking at the whole timespan of the database but still show changes in important terms like clustering and deep learning.”*

7. For fig S1 it would be helpful to add numbers on the right to indicate the total number of tools in each category.

*Thanks, we agree this is helpful for understanding the plot. Numbers have been added to the labels for each category.*

8. Fu and Hughey point out that in their dataset altmetric score and citations are only weakly correlated. Could the authors calculate this correlation for their dataset?

*We have added a new supplementary plot showing the correlation between citations and Altmetric Attention Score for publications and between total citations, total Altmetric Attention Score and GitHub stars for tools. As shown previously these are all weakly correlated with Spearman’s correlation coefficients between 0.41 and 0.66. These correlations are also mentioned in the text.*

*“Results for AAS are broadly similar to those seen for citations but it is important to note that the model fit for AAS is significantly worse than for citations. While the two metrics are correlated (Rho=0.55) (Supplementary Figure 5) AAS captures a much wider range of sources, many of which could be more highly associated with preprints than publications.”*

*“Similar to publication metrics these values are also weakly correlated but are not entirely predictive of each other (Supplementary Figure 5).”*

9. The other aspect that I was curious about the models in Fig 3 is the % of variance explained. Could you report this quantity to give a better indication of how well the models explain the data?

*Thank you for this very important point which we had previously overlooked! The model coefficient plots in Figure 3 now include an inlaid plot showing adjusted R2 values for each model. When adding these plots we realised the fits for the tools models were relatively poor compared to those for publications. This prompted us to revise the models for tools based on the fit for predicting total citations, adding new terms for whether the tool has an associated license, the number of publications, the number of preprints and the number of GitHub contributors. We replaced the previous GitHub popularity score with just the number of GitHub stars which has been used by previous studies and has been suggested as a reasonable proxy for more complex measures of GitHub popularity. These changes are described in the Methods section:*

*“We then adapted this model to consider tools rather than individual publications:*

*log2(Metric+1) Platform + Repository + License + Publications +*

*Preprints + log2(Contributors + 1) + spline(Years, df=3)*

*Here Metric is either total citations for all publications and preprints associated with a tool, total AAS for all publications and preprints or the number of GitHub stars. The Platform variable is an indicator showing whether the tool uses R, Python, both or some other platform (baseline). Repository is a boolean indicator showing whether the tool is available from any of the major software package repositories (CRAN, Bioconductor or PyPI). License is a boolean indicator variable showing whether the tool has an associated software license. Publications and Preprintsare the number of publications and preprints associated with a tool and Contributors is the number of GitHub contributors.”*

*After these changes to the model the presence of a tool in a central software repository was no longer significant for total citations and GitHub stars. This has been updated in the text as well as describing the effect of the new predictors:*

*“After accounting for the age of GitHub repositories the number of publications had the largest effect on total citations (Supplementary Table 2). Interestingly the number of preprints was a bigger predictor of total AAS suggesting that preprints attract attention and are responsible for the initial interest in a tool. The usage of Python was a significant predictor for the number of GitHub stars and the presence of a software license predicted GitHub stars and AAS while the availability of tools from a software repository was only significant for AAS.”*
